# Genome sequence of *Ensifer arboris* strain LMG 14919^T^; a microsymbiont of the legume *Prosopis chilensis* growing in Kosti, Sudan

**DOI:** 10.4056/sigs.4828625

**Published:** 2013-12-31

**Authors:** Wayne Reeve, Rui Tian, Lambert Bräu, Lynne Goodwin, Christine Munk, Chris Detter, Roxanne Tapia, Cliff Han, Konstantinos Liolios, Marcel Huntemann, Amrita Pati, Tanja Woyke, Konstantinos Mavrommatis, Victor Markowitz, Natalia Ivanova, Nikos Kyrpides, Anne Willems

**Affiliations:** 1 Centre for Rhizobium Studies, Murdoch University, Western Australia, Australia; 2 School of Life and Environmental Sciences, Deakin University, Victoria, Australia; 3 Los Alamos National Laboratory, Bioscience Division, Los Alamos, New Mexico, USA; 4 DOE Joint Genome Institute, Walnut Creek, California, USA; 5 Biological Data Management and Technology Center, Lawrence Berkeley National Laboratory, Berkeley, California, USA; 6 Laboratory of Microbiology, Department of Biochemistry and Microbiology, Faculty of Sciences, Ghent University, Belgium

**Keywords:** root-nodule bacteria, nitrogen fixation, rhizobia, *Alphaproteobacteria*

## Abstract

*Ensifer arboris* LMG 14919^T^ is an aerobic, motile, Gram-negative, non-spore-forming rod that can exist as a soil saprophyte or as a legume microsymbiont of several species of legume trees. LMG 14919^T^ was isolated in 1987 from a nodule recovered from the roots of the tree *Prosopis chilensis* growing in Kosti, Sudan. LMG 14919^T^ is highly effective at fixing nitrogen with *P. chilensis* (Chilean mesquite) and *Acacia senegal* (gum Arabic tree or gum acacia). LMG 14919^T^ does not nodulate the tree *Leucena leucocephala*, nor the herbaceous species *Macroptilium atropurpureum*, *Trifolium pratense*, *Medicago sativa*, *Lotus corniculatus* and *Galega orientalis*. Here we describe the features of *E. arboris* LMG 14919^T^, together with genome sequence information and its annotation. The 6,850,303 bp high-quality-draft genome is arranged into 7 scaffolds of 12 contigs containing 6,461 protein-coding genes and 84 RNA-only encoding genes, and is one of 100 rhizobial genomes sequenced as part of the DOE Joint Genome Institute 2010 Genomic Encyclopedia for Bacteria and Archaea-Root Nodule Bacteria (GEBA-RNB) project.

## Introduction

Legume plants form nitrogen fixing symbiosis with root nodule bacteria, collectively called rhizobia. These legumes are particularly useful crop plants that do not require exogenous nitrogenous fertilizer to support growth in less fertile, nitrogen-deficient conditions. They include some of our staple food and feed plants such as beans, peas, soybeans, lentils, clover, peanuts and alfalfa and are mostly annual crops. In many arid and savannah regions, leguminous trees represent a particularly valuable resource as they are often deep-rooted and drought resistant. They have been used traditionally in the Sahel region as sources of timber, fodder and for soil improvement [1]. *Prosopis chilensis*, also known as Chilean mesquite, is a native tree from South America that has many uses: its nutritious pods can be ground to produce flour and are also eaten by livestock; its wood is used for construction and furniture. Chilean mesquite is also used for intercropping with other plants, for which it provides shelter and nutrients (leaf compost, nitrogen). *Acacia senegal* (recently renamed as *Senegalia senegal*) is a plant of particular importance in the production of gum arabic in the Sahel region and the Middle East. Its seeds are dried for human consumption, and its leaves and pods serve as feed for sheep, goats and camels. The plant is also used in agroforestry in intercropping with watermelon and grasses, and in rotation systems with other crops (Agroforestree Database [2]).

The microsymbiont of these legume trees from Sudan and Kenya [3] has been renamed as *Ensifer arboris* [4], of which LMG 14919^T^ (= HAMBI 1552, ORS 1755, TTR38) is the type strain. This strain was isolated from root nodules of *Prosopis chilensis* from Kosti, Sudan, and shown to effectively nodulate its original host as well as *Acacia senegal* [5].

Given the drought tolerance of the host trees, it seems fitting that their symbionts are also stress resistant: *Ensifer arboris* was described as tolerant to temperatures up to 41-43 °C, 3% NaCl, several heavy metals (including Pb, Cd, Hg, Cu) and a wide range of antibiotics [3,5], characteristics that contribute to the success of the rhizobial*-*legume tree association in challenging environmental conditions [6]. Here we present a summary classification and a set of features for *E. arboris* strain LMG 14919^T^ (Table 1), together with the description of the complete genome sequence and its annotation.

**Table 1:** Classification and general features of *Ensifer arboris* LMG 14919^T^ according to the MIGS recommendations [7]

**MIGS ID**	**Property**	**Term**	**Evidence code**
	Current classification	Domain *Bacteria*	TAS [8]
		Phylum *Proteobacteria*	TAS [9]
		Class *Alphaproteobacteria*	TAS [10,11]
		Order *Rhizobiales*	TAS [11,12]
		Family *Rhizobiaceae*	TAS [13,14]
		Genus *Ensifer*	TAS [4,15,16]
		Species *Ensifer arboris*	TAS [4]
		Strain LMG 14919^T^	
	Gram stain	Negative	IDA
	Cell shape	Rod	IDA
	Motility	Motile	IDA
	Sporulation	Non-sporulating	NAS
	Temperature range	Mesophile	NAS
	Optimum temperature	28°C	NAS
	Salinity	Non-halophile	NAS
MIGS-22	Oxygen requirement	Aerobic	TAS [3]
	Carbon source	Varied	TAS [5]
	Energy source	Chemoorganotroph	NAS
MIGS-6	Habitat	Soil, root nodule, on host	TAS [3,5]
MIGS-15	Biotic relationship	Free living, symbiotic	TAS [3,5]
MIGS-14	Pathogenicity	Non-pathogenic	NAS
	Biosafety level	1	TAS [17]
	Isolation	Root nodule	TAS [5]
MIGS-4	Geographic location	Kosti, Sudan	TAS [5]
MIGS-5	Soil collection date	1987	IDA
MIGS-4.1 MIGS-4.2	Longitude Latitude	32.66342 13.16125	TAS [5] TAS [5]
MIGS-4.3	Depth	Not reported	NAS
MIGS-4.4	Altitude	Not reported	NAS

Evidence codes – IDA: Inferred from Direct Assay; TAS: Traceable Author Statement (i.e., a direct report exists in the literature); NAS: Non-traceable Author Statement (i.e., not directly observed for the living, isolated sample, but based on a generally accepted property for the species, or anecdotal evidence). These evidence codes are from the Gene Ontology project [18].

## Classification and features

**Figure 1:**
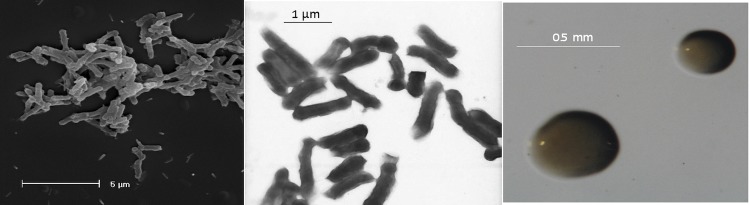
Images of *Ensifer arboris* LMG 14919^T^ using scanning (Left) and transmission (Center) electron microscopy and the appearance of colony morphology on a solid medium (Right).

*E. arboris* LMG 14919^T^ is a motile, non-sporulating, non-encapsulated, Gram-negative rod in the order *Rhizobiales* of the class *Alphaproteobacteria*. The rod-shaped form varies in size with dimensions of approximately 0.25 μm in width and 1.0-1.5 μm in length (Figure 1, Left and Center). The strain is fast-growing, forming colonies within 3-4 days when grown on half strength Lupin Agar (½LA) [19], tryptone-yeast extract agar (TY) [20] or a modified yeast-mannitol agar (YMA) [21] at 28°C. Colonies on ½LA are white-opaque, slightly domed and moderately mucoid with smooth margins (Figure 1 Right).

*E. arboris* LMG 14919^T^ is capable of using several amino acids, including L-proline, L-arginine, sodium glutamate and L-histidine as sole nitrogen sources and can use a wide range of different carbon sources including L-arabinose, D-galactose, raffinose, L-rhamnose, maltose, lactose, D-fructose, D-mannose, trehalose, D-ribose, xylene, methyl-D-mannoside, sorbitol, dulcitol, meso-inositol, inulin, dextrin, amygdalin, arbutin, sodium citrate, itaconate, α-ketoglutarate, sodium maltose, 1,2-propylene glycol, and 1,2-butylene glycol [5].

**Figure 2:**
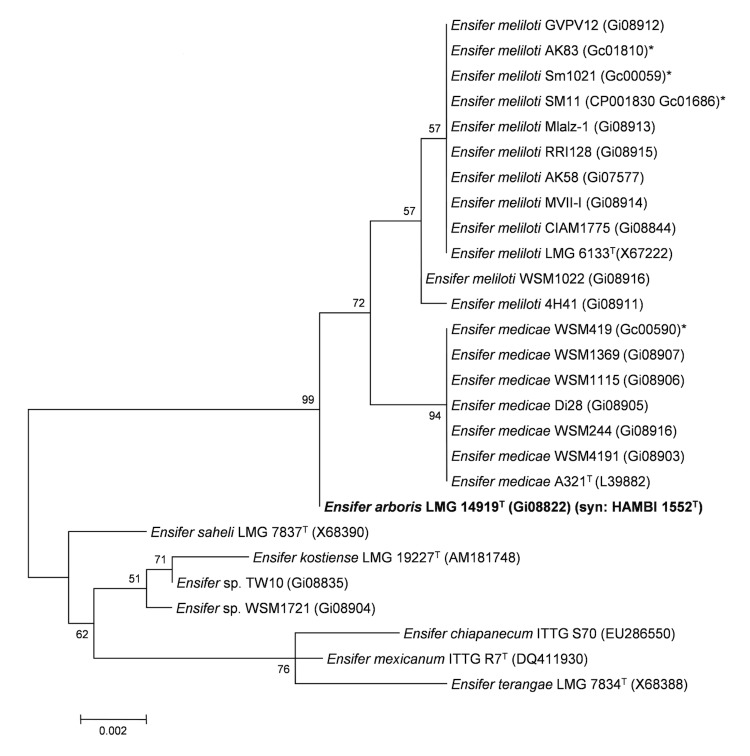
Phylogenetic tree showing the relationship of *Ensifer arboris* LMG 14919^T^ (shown in bold print) to other *Ensifer* spp. in the order *Rhizobiales* based on aligned sequences of the 16S rRNA gene (1,290 bp internal region). All sites were informative and there were no gap-containing sites. Phylogenetic analyses were performed using MEGA, version 5 [22]. The tree was built using the Maximum-Likelihood method with the General Time Reversible model [23]. Bootstrap analysis [24] with 500 replicates was performed to assess the support of the clusters. Type strains are indicated with a superscript T. Brackets after the strain name contain a DNA database accession number and/or a GOLD ID (beginning with the prefix G) for a sequencing project registered in GOLD [25]. Published genomes are indicated with an asterisk.

Minimum Information about the Genome Sequence (MIGS) is provided in Table 1. Figure 2 shows the phylogenetic neighborhood of *E. arboris* LMG 14919^T^ in a 16S rRNA sequence based tree. This strain shares 99% (1361/1366 bp) and 99% (1361/1366 bp) sequence identity to the 16S rRNA of the fully sequenced *E. meliloti* Sm1021 [26] and *E. medicae* WSM419 [27] strains, respectively.

### Symbiotaxonomy

*E. arboris* LMG 14919^T^ was initially shown to form nodules (Nod^+^) and fix nitrogen (Fix^+^) with two leguminous tree species, *P. chilensis* and *A. senegal.* It was unable to elicit nodules on the herbaceous perennials *Macroptilium atropurpureum*, *Trifolium pratense*, *Medicago sativa*, *Lotus corniculatus* and *Galega orientalis* [5]. The symbiotic properties of this strain in seedlings of *Acacia* and *Prosopis* spp. in Sudan and Senegal have been reported in detail [6]. Indeterminate nodules are induced, mainly on the lateral roots either in clusters or individually. Young nodules are spherical and later become elongated and are commonly branched. LMG 14919^T^ (=HAMBI 1552) was shown to nodulate and fix nitrogen in seedlings of African *A. mellifera*, *A. nilotica*, *A. oerfota* (synonym *A. nubica*), *A. senegal*, *A. seyal*, *A. sieberiana*, *A. tortilis* subsp. *raddiana*, Latin American *A. angustissima*, *P. chilensis* and *P. pallida*, and Afro-Asian *P. cineraria*. It also effectively nodulates with Latin-American introductions of *P. chilensis* and *P. juliflora* in Africa [6]. It induced small ineffective nodules on Australian *A. holosericea* and African *P. africana* [6].

## Genome sequencing and annotation

### Genome project history

This organism was selected for sequencing on the basis of its environmental and agricultural relevance to issues in global carbon cycling, alternative energy production, and biogeochemical importance, and is part of the Community Sequencing Program at the U.S. Department of Energy, Joint Genome Institute (JGI) for projects of relevance to agency missions. The genome project is deposited in the Genomes OnLine Database [25] and an improved-high-quality-draft genome sequence in IMG. Sequencing, finishing and annotation were performed by the JGI. A summary of the project information is shown in Table 2.

**Table 2:** Genome sequencing project information for *E. arboris* LMG 14919^T^.

**MIGS ID**	**Property**	**Term**
MIGS-31	Finishing quality	Improved high-quality draft
MIGS-28	Libraries used	Illumina Standard (short PE) and Illumina CLIP (long PE) library
MIGS-29	Sequencing platforms	Illumina HiSeq 2000
MIGS-31.2	Sequencing coverage	Illumina: 448x
MIGS-30	Assemblers	Velvet version 1.1.05; Allpaths-LG version r38445
MIGS-32	Gene calling methods	Prodigal 1.4, GenePRIMP
	GenBank	ATYB00000000
	GenBank release date	July 15, 2013
	GOLD ID	Gi08822
	NCBI project ID	74465
	Database: IMG	2512047086
	Project relevance	Symbiotic N_2_ fixation, agriculture

### Growth conditions and DNA isolation

*E. arboris* LMG 14919^T^ was cultured to mid logarithmic phase in 60 ml of TY rich medium on a gyratory shaker at 28°C [28]. DNA was isolated from the cells using a CTAB (Cetyl trimethyl ammonium bromide) bacterial genomic DNA isolation method [29].

### Genome sequencing and assembly

The genome of *Ensifer arboris* LMG 14919^T^ was sequenced at the Joint Genome Institute (JGI) using Illumina technology [30]. An Illumina short-insert paired-end library with an average insert size of 270 bp generated 19,256,666 reads and an Illumina long-insert paired-end library with an average insert size of 9,232.94 +/- 2,530.88 bp generated 1,365,298 reads totaling 3,093.3 Mbp of Illumina data. All general aspects of library construction and sequencing performed at the JGI can be found at the JGI user home.

The initial draft assembly contained 27 contigs in 9 scaffolds. The initial draft data was assembled with Allpaths, version r38445, and the consensus was computationally shredded into 10 Kbp overlapping fake reads (shreds). The Illumina draft data was also assembled with Velvet, version 1.1.05 [31], and the consensus sequences were computationally shredded into 1.5 Kbp overlapping fake reads (shreds). The Illumina draft data was assembled again with Velvet using the shreds from the first Velvet assembly to guide the next assembly. The consensus from the second VELVET assembly was shredded into 1.5 Kbp overlapping fake reads. The fake reads from the Allpaths assembly and both Velvet assemblies and a subset of the Illumina CLIP paired-end reads were assembled using parallel phrap, version SPS 4.24 (High Performance Software, LLC). Possible mis-assemblies were corrected with manual editing in Consed [32-34]. Gap closure was accomplished using repeat resolution software (Wei Gu, unpublished), and sequencing of bridging PCR fragments using Sanger (unpublished, Cliff Han) technology. For the improved high quality draft, one round of manual/wet lab finishing was completed. A total of 46 additional sequencing reactions, were completed to close gaps and to raise the quality of the final sequence. The estimated total size of the genome is 6.9 Mbp and the final assembly is based on 3,093.3 Mbp of Illumina draft data, which provides an average of 448× coverage of the genome.

### Genome annotation

Genes were identified using Prodigal [35] as part of the DOE-JGI annotation pipeline [36] followed by a round of manual curation using the JGI GenePRIMP pipeline [37]. The predicted CDSs were translated and used to search the National Center for Biotechnology Information (NCBI) non-redundant database, UniProt, TIGRFam, Pfam, PRIAM, KEGG, COG, and InterPro databases. These data sources were combined to assert a product description for each predicted protein. Non-protein coding genes and miscellaneous features were predicted using tRNAscan-SE [38], RNAMMer [39], searches against models of the ribosomal RNA genes built from SILVA [40], Rfam [41], TMHMM [42], and SignalP [43]. Additional gene prediction analysis and manual functional annotation was performed within the Integrated Microbial Genomes (IMG-ER) platform [44].

## Genome properties

**Figure 3:**
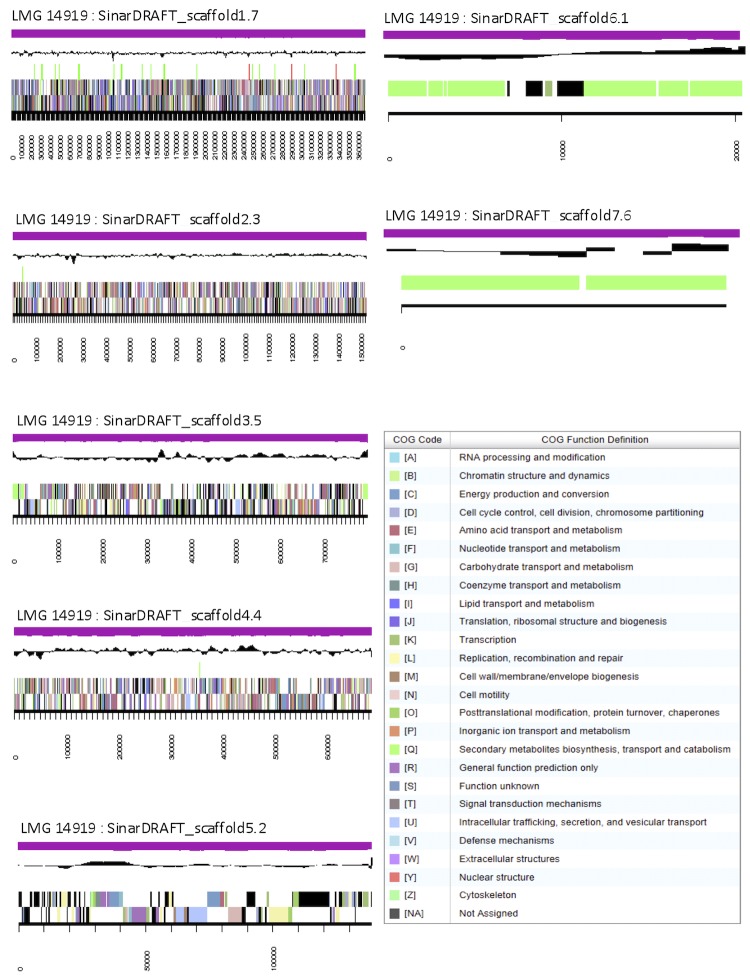
Graphical map of the genome of *Ensifer arboris* LMG 14919^T^ showing the seven largest scaffolds. From bottom to the top of each scaffold: Genes on forward strand (color by COG categories as denoted by the IMG platform), Genes on reverse strand (color by COG categories), RNA genes (tRNAs green, sRNAs red, other RNAs black), GC content, GC skew.

The genome is 6,850,303 nucleotides with 62.02% GC content (Table 3) and comprised of 7 scaffolds (Figure 3) of 12 contigs. From a total of 6,545 genes, 6,461 were protein encoding and 84 RNA only encoding genes. The majority of genes (80.78%) were assigned a putative function whilst the remaining genes were annotated as hypothetical. The distribution of genes into COGs functional categories is presented in Table 4.

**Table 3:** Genome Statistics for *Ensifer arboris* LMG 14919^T^

**Attribute**	**Value**	**% of Total**
Genome size (bp)	6,850,303	100.00
DNA coding region (bp)	5,921,899	86.45
DNA G+C content (bp)	4,248,771	62.02
Number of scaffolds	7	
Number of contigs	12	
Total gene	6,545	100.00
RNA genes	84	1.28
rRNA operons	3	0.05
Protein-coding genes	6,461	98.72
Genes with function prediction	5,287	80.78
Genes assigned to COGs	5,233	79.95
Genes assigned Pfam domains	5,438	83.09
Genes with signal peptides	588	8.98
Genes with transmembrane helices	1,456	22.25
CRISPR repeats	0	

**Table 4:** Number of protein coding genes of *Ensifer arboris* LMG 14919^T^ associated with the general COG functional categories.

**Code**	**Value**	**% age**	**Description**
J	195	3.35	Translation, ribosomal structure and biogenesis
A	0	0.00	RNA processing and modification
K	510	8.76	Transcription
L	212	3.64	Replication, recombination and repair
B	1	0.02	Chromatin structure and dynamics
D	49	0.84	Cell cycle control, mitosis and meiosis
Y	0	0.00	Nuclear structure
V	60	1.03	Defense mechanisms
T	248	4.26	Signal transduction mechanisms
M	274	4.71	Cell wall/membrane biogenesis
N	77	1.32	Cell motility
Z	0	0.00	Cytoskeleton
W	0	0.00	Extracellular structures
U	122	2.10	Intracellular trafficking and secretion
O	185	3.18	Posttranslational modification, protein turnover, chaperones
C	349	6.00	Energy production conversion
G	598	10.27	Carbohydrate transport and metabolism
E	653	11.22	Amino acid transport metabolism
F	104	1.79	Nucleotide transport and metabolism
H	201	3.45	Coenzyme transport and metabolism
I	205	3.52	Lipid transport and metabolism
P	292	5.02	Inorganic ion transport and metabolism
Q	182	3.13	Secondary metabolite biosynthesis, transport and catabolism
R	721	12.39	General function prediction only
S	582	10.00	Function unknown
-	1,312	20.05	Not in COGS
